# Supplementary material for: Nutrient Supply Is Essential for Shifting Tree Peony Reflowering Ahead in Autumn and Sugar Signaling Is Involved
Source: Int J Mol Sci. 2022 Jul 12;23(14):7703. doi: 10.3390/ijms23147703 (PMC9315773; doi:10.3390/ijms23147703)
Supplement: Supplementary file 1 [file ijms-23-07703-s001.zip › ijms-1781988-supplementary.pdf]

Table S1. Statistical analysis of bud development and flowering

Treatments	Bud diameter (mm)	New branch length (cm)	Max leaf length (cm)	Flowering rate (%)	Initial flowering time
**Control**	20.40±0.15 a	19.93±0.34 b	15.43±0.69 b	81.86 a	October 8th
**GA treatment**	9.92±0.21 c	33.10±0.78 a	18.80±3.70 ab	0.00 c	n/a
**GA+Nutrient**	14.64±0.52 b	31.71±0.33 a	20.56±1.08 a	62.57 b	August 29th

Note: error bars indicate the standard deviation (n = 5), and different lowercase letters indicate significant differences (Tukey's test, α = 0.05). n/a, not apply.

Table S2. Effect of various fertilizers on reflowering of tree peony in autumn

Fertilizer type	The ratio of N, P, K	Flower diameter (cm)	New branch length (cm)	Initial flowering period
Control	0:0:0	12.83±1.19b	22.32±1.59a	Sep. 23, 2019
Type I	1:1:1	15.86±1.27a	23.41±2.47a	Sep. 22, 2019
Type II	1:3:2	15.58±1.41a	22.77±1.75a	Sep. 19, 2019
Type III	3:2:6	15.34±1.13a	23.18±1.66a	Sep. 23, 2019

Table S3. The primer sequence information used for gene expression in this study

Gene name	Primer name	Sequence (5'-3')	Gene ID
PsAP1	RTAP1-F	AGAAGAAGGAAAGGGCAATC	KF113361.1
	RTAP1-R	TTCCTCCTCACTTCTGTTGG	
PsFT	RTFT-F	CCAAGCGACCCAAACCTA	KF113360.1
	RTFT-R	CGCCAACCTGGAGTGTAA	
PsLFY	RTLFY-F	ATGAGAAGGAAGGAGGGGATG	psu.G.00032564
	RTLFY-R	CTTTGGCAATGGTCTGAACT	
PsSOC1	RTSOC1-F	CCAATGTCCGAGCAAGAAAG	KR779924.1
	RTSOC1-R	CCGTGCTTCTCGCATAACAT	
PsSVP	RTSVP-F	CGATGTTGAGCAAGGAGGTT	KC847164.1
	RTSVP-R	GCTCTAAATCAGCAGCGACA	
PsGA20ox	RTGA20ox-F	TGGGTGGTCTTCAAGTGTTT	MH546118.1
	RTGA20ox-R	AGCCCTGTAAATGTTTCTGTG	
PsGA3ox	RTGA3ox-F	TGATGTGGTTGATGTTGGGCT	MH546120.1
	RTGA3ox-R	GAGAAGAGTTGAATCTGTGTGTGC	
PsGA2ox	RTGA2ox-F	CTCCAAACCCGACACTAAACA	MH546119
	RTGA2ox-R	GCCCAATGTTCTTGTTCCCA	
PsGID1c	RTGID1c-F	TGAAGAACCTCCACCAAG	MH546122
	RTGID1c-R	CCACAAGACGACGACAAA	
PsGAI	RTGAI-F	GAGTATGCTGTCCGAGTTCA	MH550803
	RTGAI-R	CAGGAGCAAGGAACGAAT	
PsHXK1	RTHXK1-F	ATAAGAAAAGCCGTGGTAGAGC	MT136704
	RTHXK1-R	AGTGTTCATACAAGCCACCATC	
PsSnRK1	RTSnRK1-F	CACGAAGGAATGGCGAATA	MT136703
	RTSnRK1-R	CAAGGAAGGCAGCACAAAG	
PsTPS1	RTTPS1-F	CTCTCGACTTCCATGCCTTA	MT136702
	RTTPS1-R	CTGTGATGTTCCAAGGGTTC	
Actin	Actin-F	GAGAGATTCCGTTGCCCAG	JN105298
	Actin-R	TCCTTGCTCATTCTGTCTGC	

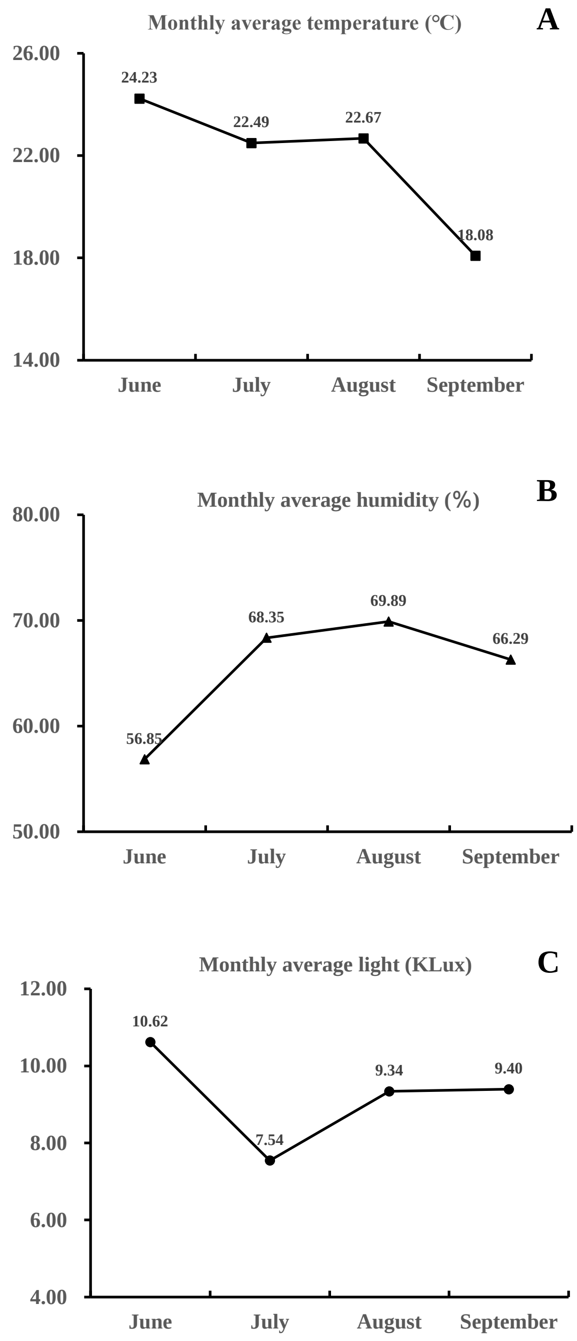

Figure S1. Environmental conditions in the process of tree peony 'Qiu Fa No. 1' bud growth and development. A: Monthly average temperature; B: Monthly average humidity; C: Monthly average light.
